# Supplementary material for: Agrobacterium expressing a type III secretion system delivers Pseudomonas effectors into plant cells to enhance transformation
Source: Nat Commun. 2022 May 11;13:2581. doi: 10.1038/s41467-022-30180-3 (PMC9095702; doi:10.1038/s41467-022-30180-3)
Supplement: Supplementary file 1 — Supplementary Information [file 41467_2022_30180_MOESM1_ESM.pdf]

***Agrobacterium* expressing type III secretion system delivers *Pseudomonas* effectors into plant cells to enhance transformation**

Raman *et al.*

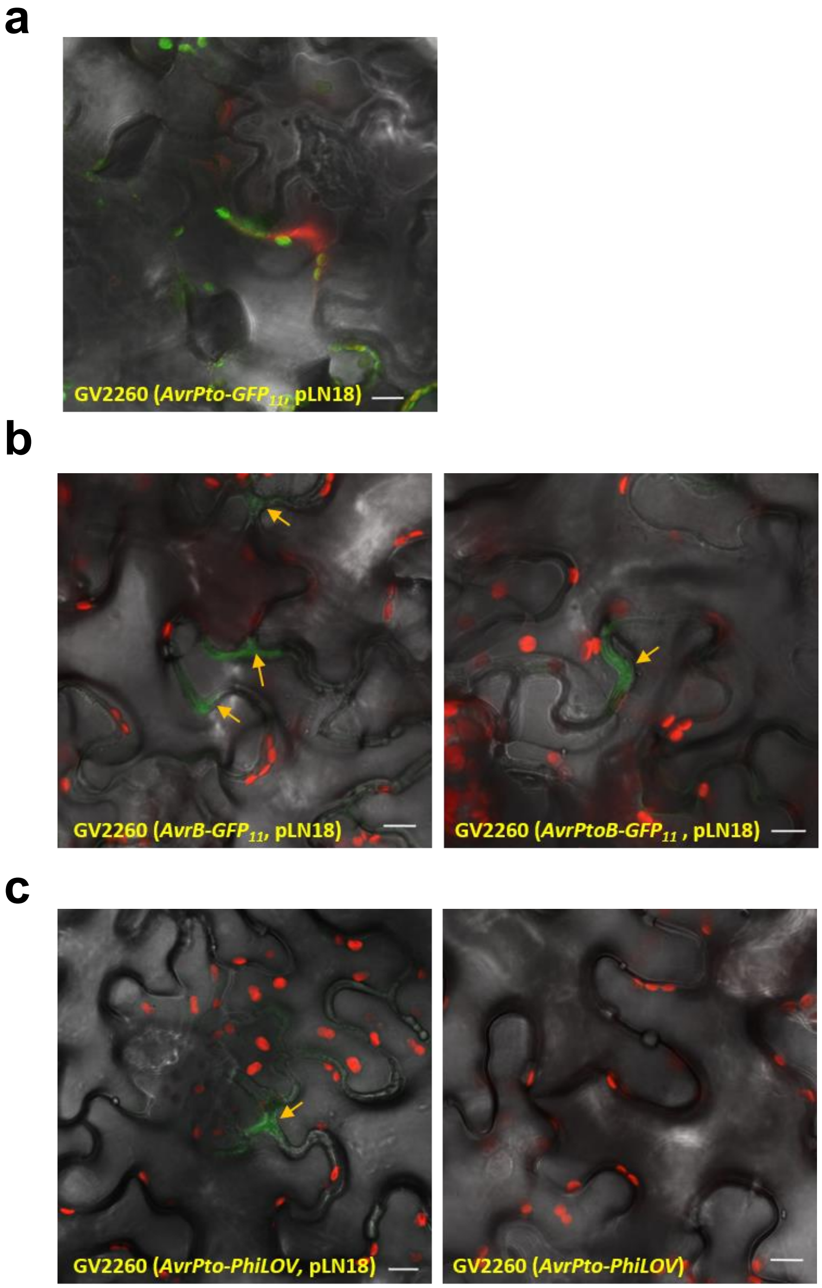

Supplementary Figure 1. *Pseudomonas* T3SS expressed in *A. tumefaciens* delivers T3Es into plant cells. (**a**) Plasma membrane localization of AvrPto. *A. tumefaciens* GV2260 (*AvrPto-GFP*$_{11}$, pLN18) (A_{600} = 0.4) infiltrated into *N. benthamiana* leaves transiently expressing *GFP*$_{1\text{-}10}$ shows plasma membrane localization of AvrPto. FM4-64 dye was infiltrated 1 h prior to taking confocal images to stain the plasma membrane. Confocal microscopy was used to visualize fluorescence 48 h post-infiltration of *A. tumefaciens*. GFP signals and chlorophyll autofluorescence were pseudo-colored to green, and FM4-64 is shown in red. (**b**) *In planta* visualization of *GFP*$_{11}$ tagged T3Es. *N. benthamiana* leaves transiently expressing *GFP*$_{1\text{-}10}$ were infiltrated with *A. tumefaciens* strains GV2260 (*AvrB-GFP*$_{11}$, pLN18) or GV2260 (*AvrPtoB-GFP*$_{11}$, pLN18) (A_{600} = 0.4). Confocal microscopy was used to visualize GFP fluorescence 48 h post-infiltration. GFP signals were pseudo-colored to green and chlorophyll autofluorescence is shown in red. (**c**) *In planta* visualization of PhiLOV tagged AvrPto. *N. benthamiana* leaves were infiltrated with *A. tumefaciens* strain GV2260 (*AvrPto-PhiLOV*, pLN18) (A_{600} = 0.4). *A. tumefaciens* strain GV2260 (*AvrPto-PhiLOV*) was used as a negative control. Confocal microscopy was used to visualize PhiLOV fluorescence 48 h post-infiltration. PhiLOV signals were pseudo-colored to green and chlorophyll autofluorescence is shown in red. Scale bars, 10 µm (a-c). Experiments were repeated two times with similar results.

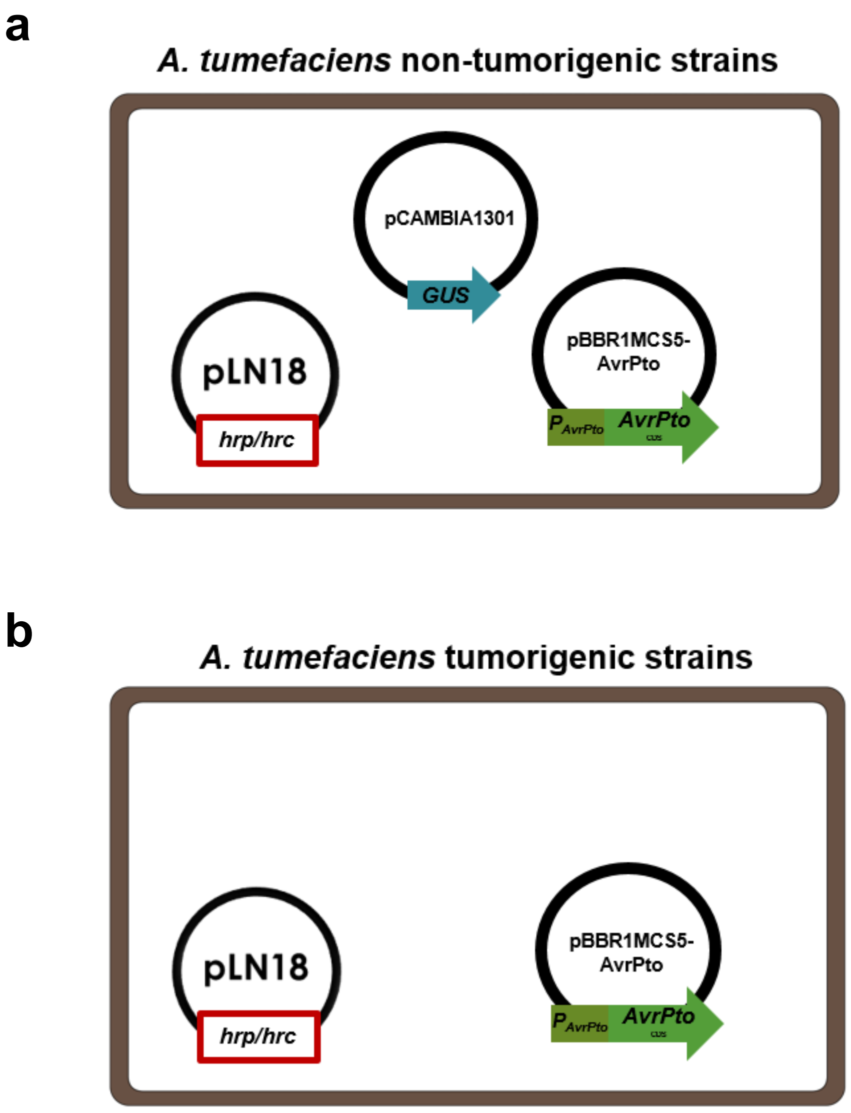

Supplementary Figure 2. Schematics of engineering *A. tumefaciens* to deliver proteins through a T3SS for transformation assays. (**a**) For transient transformation assays, the non-tumorigenic *A. tumefaciens* strains EHA105 or GV2260 harboring a binary vector pCAMBIA1301 were mobilized with the plasmids pLN18 and pBBR1MCS5-AvrPto to express a T3SS and *AvrPto*. An intron-interrupted *GUS* gene within the T-DNA of pCAMBIA1301 enables *in planta* expression of *GUS* and prevents its expression in *A. tumefaciens*. (**b**) For stable transformation assays, the tumorigenic *A. tumefaciens* strains A208 and A348 were mobilized with plasmids the pLN18 and pBBR1MCS5-AvrPto to express a T3SS and *AvrPto.*

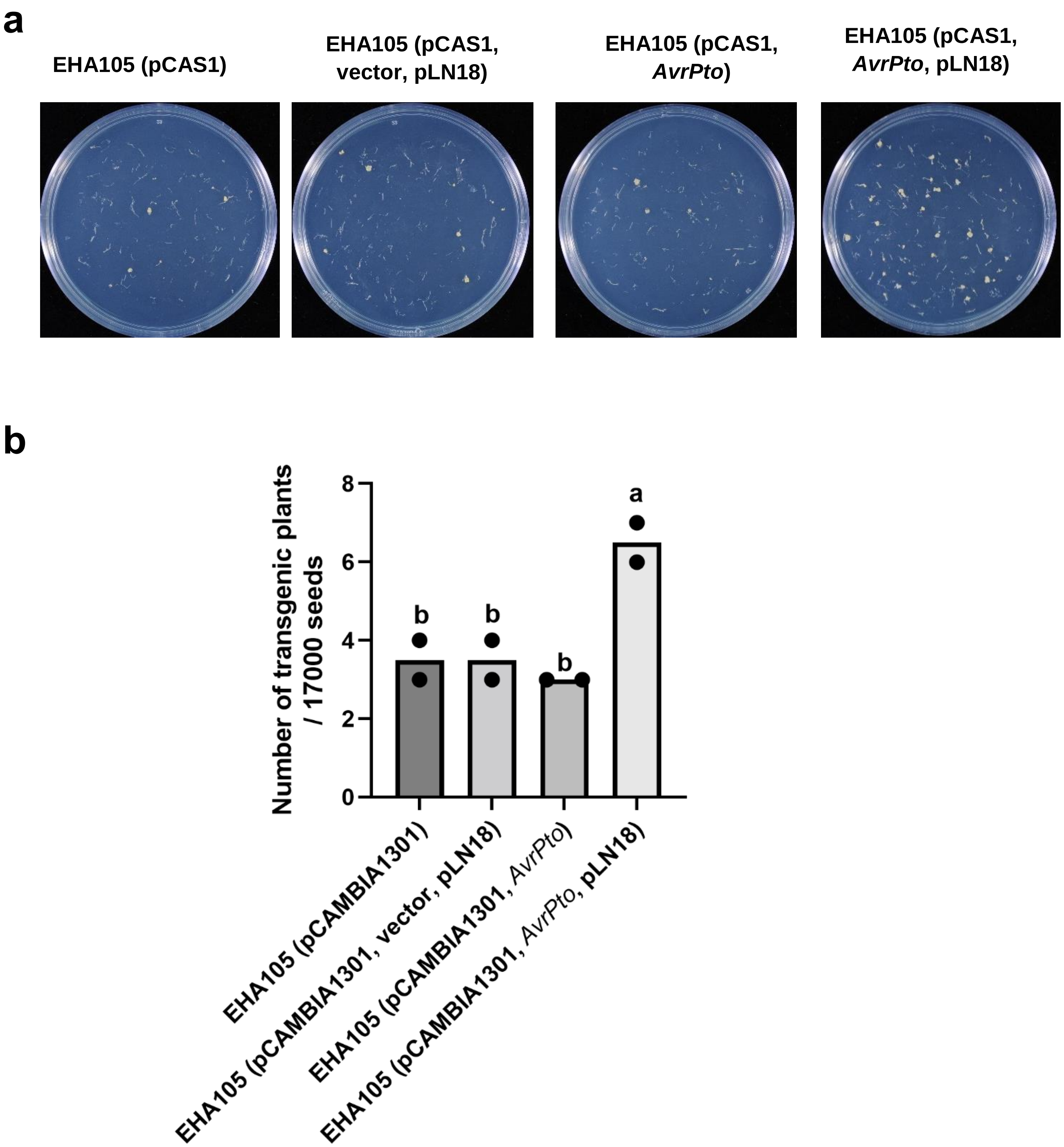

Supplementary Figure 3. Engineered *A. tumefaciens* strains expressing a T3SS and *AvrPto* greatly increase the stable transformation in Arabidopsis. (**a**) Root callus assay. Representative images of root segments forming phosphinothricin (PPT)-resistant calli for the data presented in Figure 2e. (**b**) Floral dip transformation assay. Data presented are the number of hygromycin resistant and GUS positive transgenic plants germinated from 17,000 T0 seeds in two independent experiments as mean. Bars with different letters are significantly different according to Tukey's post-hoc two-way ANOVA test (P = 0.0389). Source data are provided as a Source Data file.

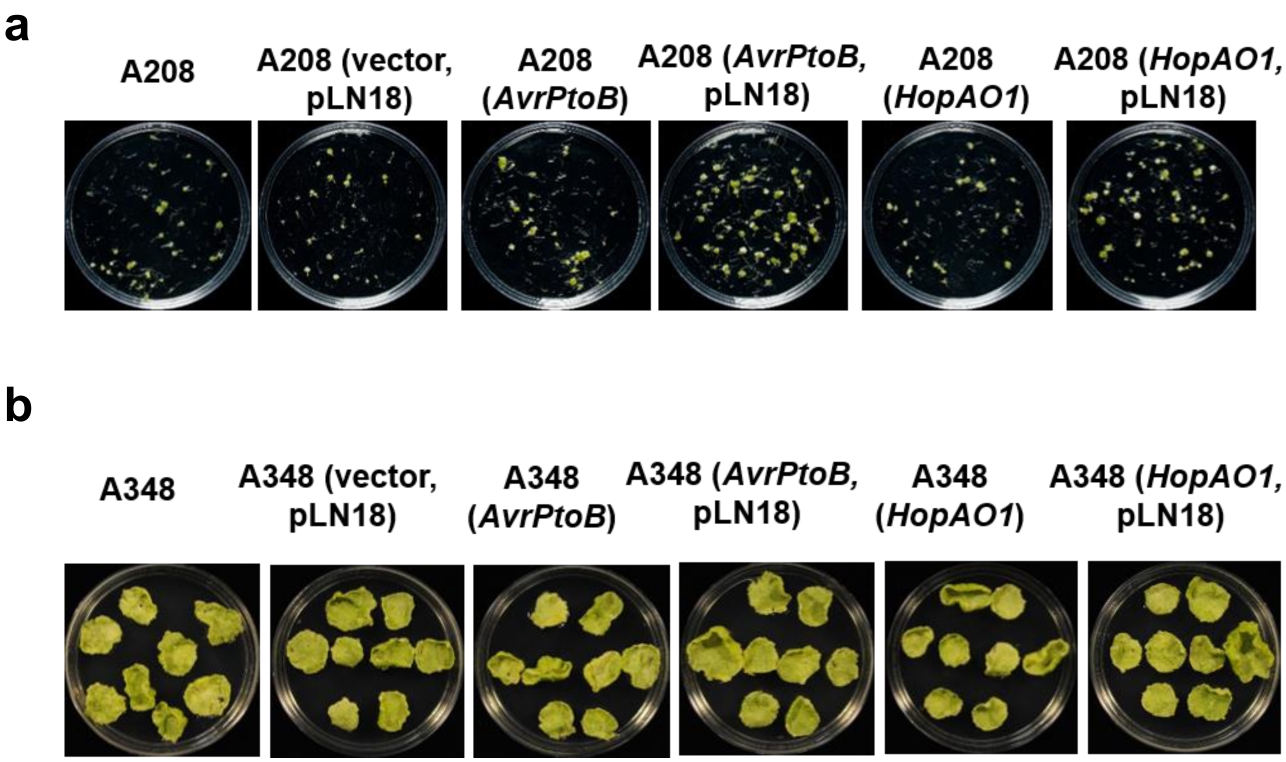

Supplementary Figure 4. Engineered *A. tumefaciens* strains expressing a T3SS and *AvrPtoB* or *HopAO1* greatly increase the stable transformation efficiency of Arabidopsis and *N. benthamiana*. (**a**) Root tumor assay. Representative images of root segments forming tumors taken 4 weeks after *A. tumefaciens* infection for the data presented in Figure 4a. (**b**) Leaf disk transformation assay. Representative images taken 10 days after *A. tumefaciens* infection for the data presented in Figure 4b.

a

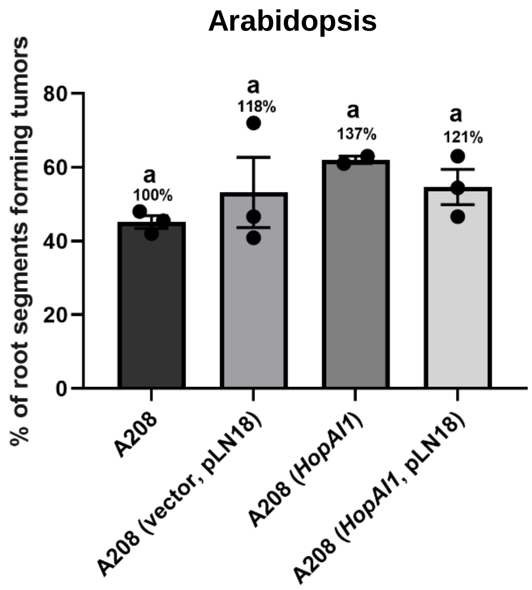

b

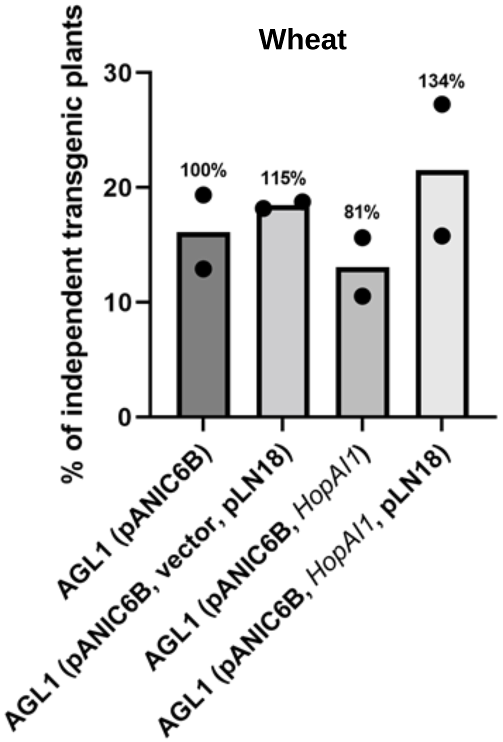

Supplementary Figure 5. Engineered *A. tumefaciens* strains expressing a T3SS and *HopAI1* did not increase the stable transformation in Arabidopsis and wheat. (**a**) Arabidopsis root tumor and (**b**) wheat transformation assays and data analysis were carried out as described in Figure 2d and Figure 5a respectively using *A. tumefaciens* strains expressing *HopAI1* and T3SS. Negative controls were also included. Source Data are provided as a Source Data file.

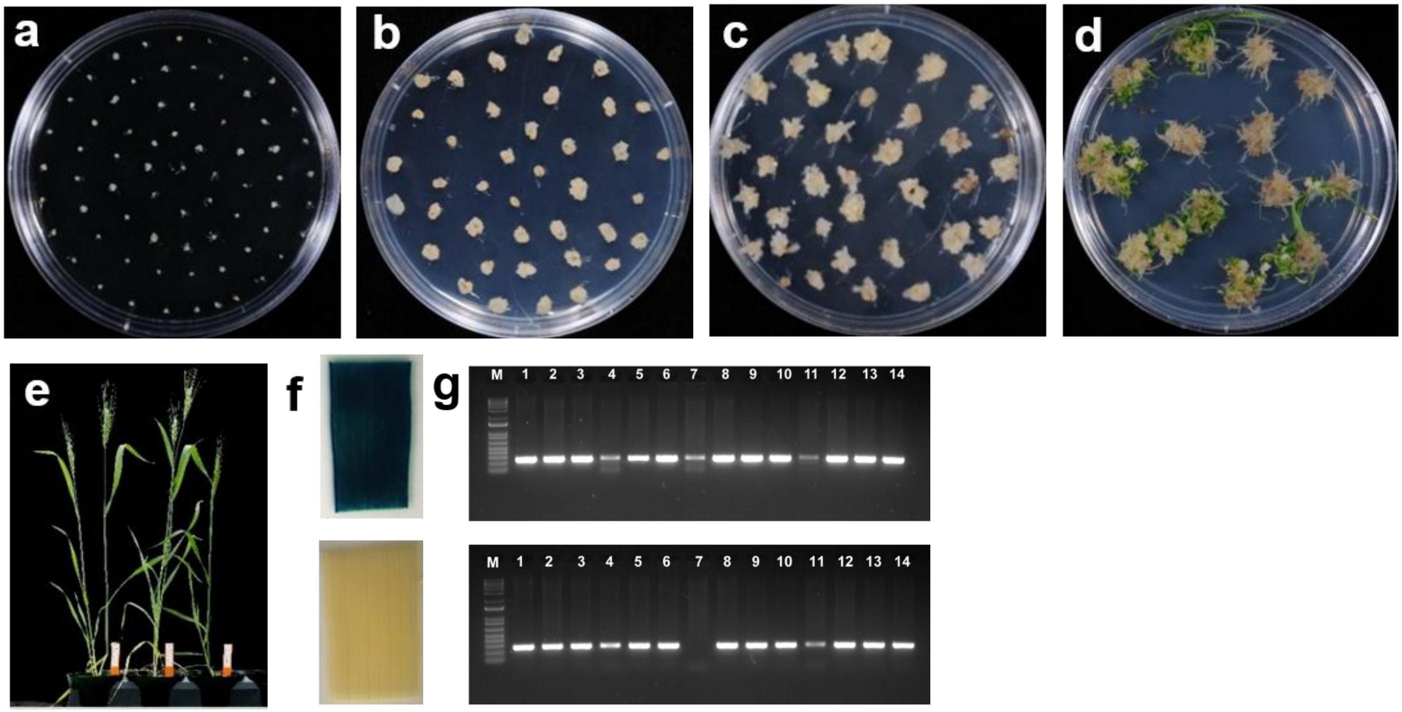

Supplementary Figure 6. Different stages of *Agrobacterium*-mediated wheat transformation. Representative images from various stages of the wheat transformation process are shown. (**a**) Freshly infected immature embryos on co-cultivation medium, (**b**) infected immature embryos on the first selection medium at 3 weeks, (**c**) second selection medium at 5 weeks and (**d**) shoot regeneration medium at 8 weeks, (**e**) transgenic wheat plants at 6 weeks after transfer to soil in the green house, (**f**) GUS stained leaf segments of transgenic wheat (top panel) and wild type wheat (bottom panel), and (**g**) PCR gels using *GUSPlus* (top panel) and *hph* (bottom panel) specific primers for confirmation of transgenic plants. M, 1kb plus ladder. Source data are provided as a Source Data file.

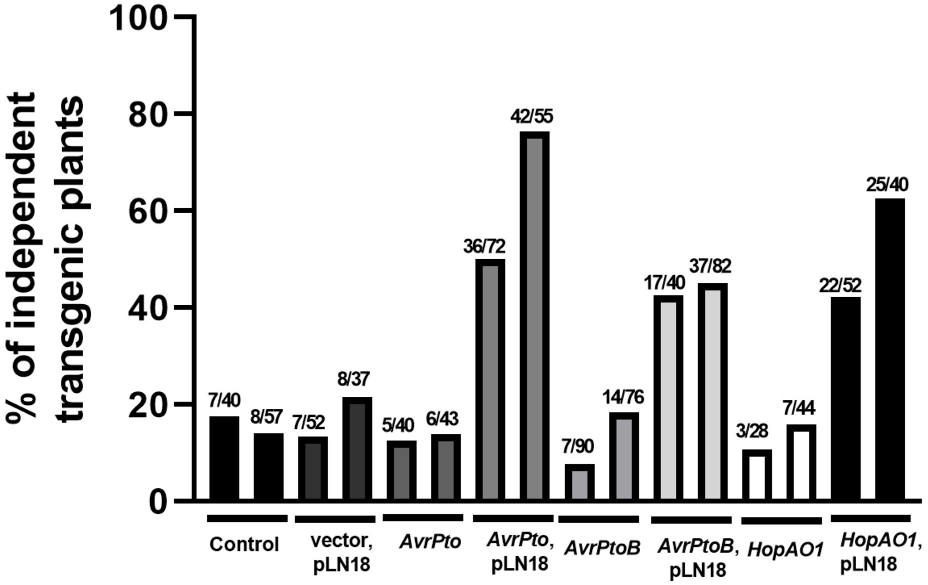

Supplementary Figure 7. *A. tumefaciens* expressing T3SS and T3Es modulate stable transformation in wheat. Immature embryos of wheat were infected with AGL-1 (pANIC6B) (designated as control in this figure) expressing pLN18 and T3Es *AvrPto*, *AvrPtoB* or *HopAO1*. AGL-1 (pANIC6B) alone, vector and pLN18, and *AvrPto*, *AvrPtoB* or *HopAO1* were included as negative controls. Number of independent transgenic plants were counted. Data presented are % of independent transgenic plants from two independent experiments shown in Figure 5. Fractions above the histogram bars indicate the number of independent transgenic plants (numerator) and the number of immature embryos used (denominator).

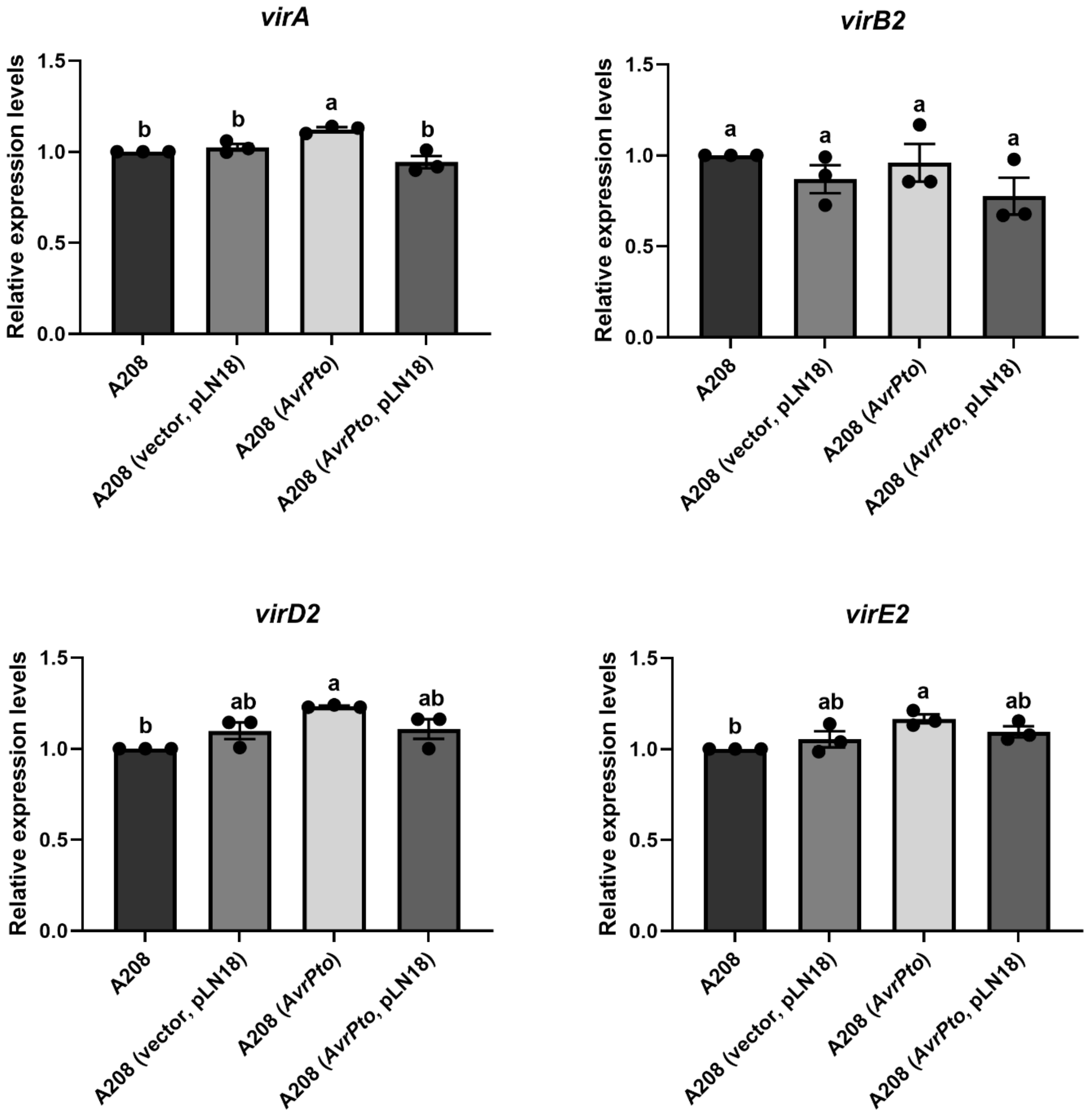

Supplementary Figure 8. *vir* gene expression of *A. tumefaciens* strains expressing a T3SS and *AvrPto*. Relative expression levels of *vir* genes. The tumorigenic *A. tumefaciens* strain A208 expressing *AvrPto* in combination with and without pLN18 or A208 alone or A208 (vector, pLN18) were grown in AB-MES medium for 24 h. Expression levels of *virA*, *virB2*, *virD2* and *virE2* were determined using $2^{-\Delta\Delta CT}$ method with the *recA* gene as a housekeeping control. *vir* gene expression values in *A. tumefaciens* strain A208 were arbitrarily set to 1. Data presented are mean ± standard error of three replicates. Bars with different letters are significantly different according to Tukey's post-hoc one-way ANOVA test (one-sided) (p = 0.0004 for *virA,* 0.2930 for *virB2,* 0.0023 for *virD2,* 0.0080 for *virE2*). Experiments were repeated three times with similar results. Source Data are provided as a Source Data file.

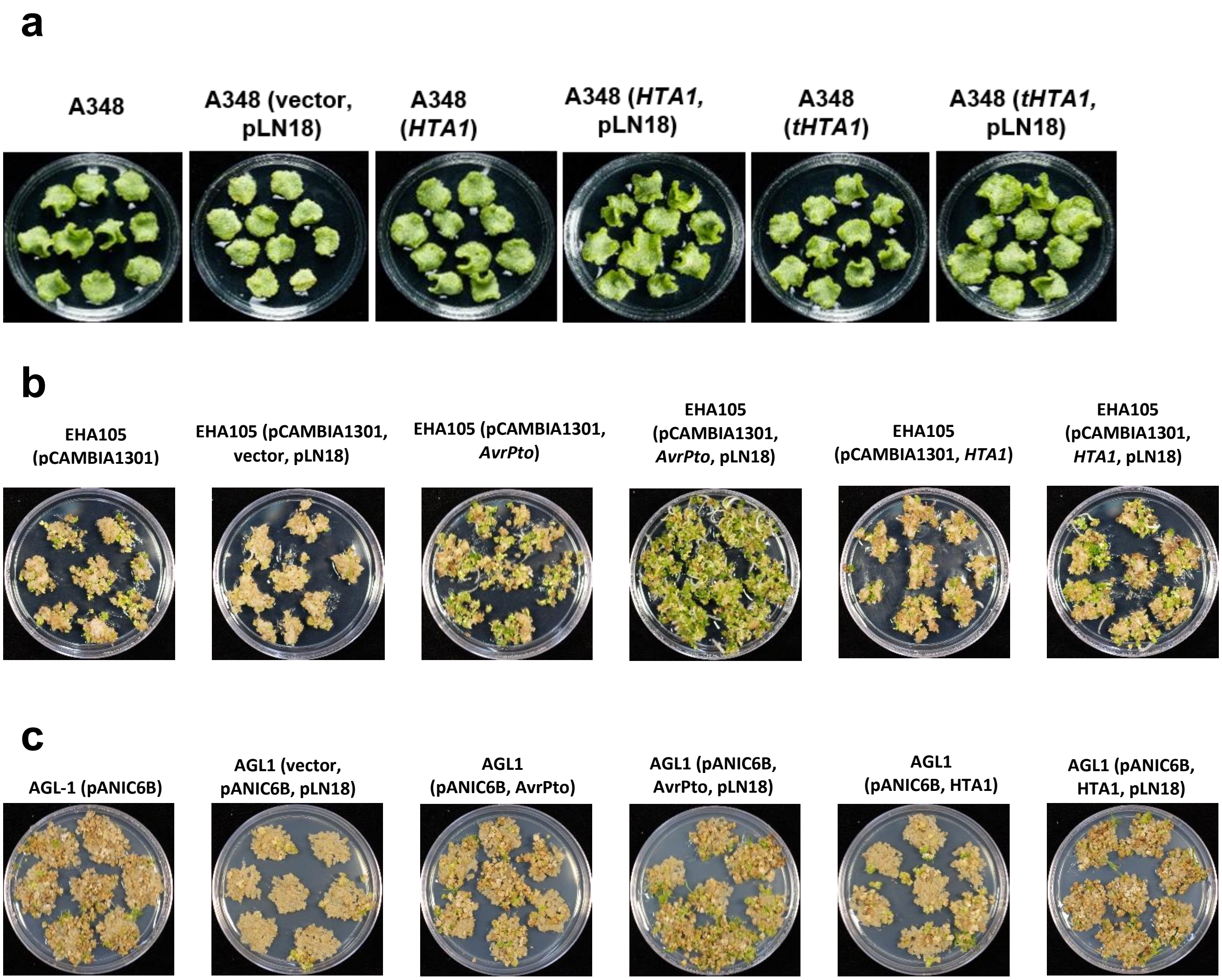

Supplementary Figure 9. Engineered *A. tumefaciens* strains expressing a T3SS and *AvrPto* or *HTA1* greatly increase the stable transformation efficiency. (**a**) *N. benthamiana* leaf disk transformation assay. Representative images taken 10 days after *A. tumefaciens* infection for the data presented in Figure 7c. (**b**) Alfalfa transformation. Representative images from plates containing shoots on regeneration medium for the data presented in Figures 5b and 7e. (**c**) Switchgrass transformation. Representative images from plates containing shoots on regeneration medium for the data presented in Figures 5c and 7f.
