# Supplementary material for: Agrobacterium expressing a type III secretion system delivers Pseudomonas effectors into plant cells to enhance transformation
Source: Nat Commun. 2022 May 11;13:2581. doi: 10.1038/s41467-022-30180-3 (PMC9095702; doi:10.1038/s41467-022-30180-3)
Supplement: Supplementary file 3 — Description of Additional Supplementary Files [file 41467_2022_30180_MOESM3_ESM.pdf]

Title: Supplementary Data 1.

Description: Plasmids and bacterial strains used in this study.

Title: Supplementary Data 2.

Description: Primers used in this study.
