# Supplementary material for: Agrobacterium expressing a type III secretion system delivers Pseudomonas effectors into plant cells to enhance transformation
Source: Nat Commun. 2022 May 11;13:2581. doi: 10.1038/s41467-022-30180-3 (PMC9095702; doi:10.1038/s41467-022-30180-3)
Supplement: Supplementary file 6 — Reporting Summary [file 41467_2022_30180_MOESM6_ESM.pdf]

nature portfolio

Corresponding author(s): Kirankumar Mysore

Last updated by author(s): Apr 7, 2022

Reporting Summary

Nature Portfolio wishes to improve the reproducibility of the work that we publish. This form provides structure for consistency and transparency in reporting. For further information on Nature Portfolio policies, see our Editorial Policies and the Editorial Policy Checklist.

Statistics

For all statistical analyses, confirm that the following items are present in the figure legend, table legend, main text, or Methods section.

n/a	Confirmed	
☐	☒	The exact sample size (*n*) for each experimental group/condition, given as a discrete number and unit of measurement
☐	☒	A statement on whether measurements were taken from distinct samples or whether the same sample was measured repeatedly
☐	☒	The statistical test(s) used AND whether they are one- or two-sided<br>*Only common tests should be described solely by name; describe more complex techniques in the Methods section.*
☒	☐	A description of all covariates tested
☒	☐	A description of any assumptions or corrections, such as tests of normality and adjustment for multiple comparisons
☐	☒	A full description of the statistical parameters including central tendency (e.g. means) or other basic estimates (e.g. regression coefficient) AND variation (e.g. standard deviation) or associated estimates of uncertainty (e.g. confidence intervals)
☐	☒	For null hypothesis testing, the test statistic (e.g. *F*, *t*, *r*) with confidence intervals, effect sizes, degrees of freedom and *P* value noted<br>*Give P values as exact values whenever suitable.*
☒	☐	For Bayesian analysis, information on the choice of priors and Markov chain Monte Carlo settings
☒	☐	For hierarchical and complex designs, identification of the appropriate level for tests and full reporting of outcomes
☒	☐	Estimates of effect sizes (e.g. Cohen's *d*, Pearson's *r*), indicating how they were calculated

Our web collection on statistics for biologists contains articles on many of the points above.

Software and code

Policy information about availability of computer code

Data collection	Confocal micrographs were acquired in a Leica SP8 confocal microscope using Leica Application Suite X (LAS X) software version 3.5.5.19976. Fluorescence intensity were measured in a Tecan Infinite® 200 Pro multimode plate reader using i-control version 11 software. qPCR data was collected using Bio-Rad CFX Manager Version 2.1.1022.0523 software.
Data analysis	GraphPad Prism version 8.0.1 is used for making graphs and ANOVA test. R version 3.5.2 is used for all Tukey's post-hoc tests.

For manuscripts utilizing custom algorithms or software that are central to the research but not yet described in published literature, software must be made available to editors and reviewers. We strongly encourage code deposition in a community repository (e.g. GitHub). See the Nature Portfolio guidelines for submitting code & software for further information.

Data

Policy information about availability of data

All manuscripts must include a data availability statement. This statement should provide the following information, where applicable:

- Accession codes, unique identifiers, or web links for publicly available datasets
- A description of any restrictions on data availability
- For clinical datasets or third party data, please ensure that the statement adheres to our policy

Data availability

All relevant data supporting the key findings of this study are available within the article and its Supplementary Information files. Source data for Figures 1b, 2b, 2d, 2e, 3b, 3d, 4, 5, 6, 7b-f and Supplementary Figures 3, 5, 6g, 8 are provided as a Source Data file.

Field-specific reporting

Please select the one below that is the best fit for your research. If you are not sure, read the appropriate sections before making your selection.

[x] Life sciences [ ] Behavioural & social sciences [ ] Ecological, evolutionary & environmental sciences

For a reference copy of the document with all sections, see nature.com/documents/nr-reporting-summary-flat.pdf

Life sciences study design

All studies must disclose on these points even when the disclosure is negative.

Sample size	Sample sizes were chosen based on similar studies published previously. For Arabidopsis root tumor and callus assays, minimum 60 root segments per plate (i.e., replicate) were analyzed (based on Gelvin 2006, reference 33 in the manuscript). For Arabidopsis leaf transient assays, five leaves per plant were infiltrated and pooled for each replicate (based on Cana-Quijada et al. 2018 Bio-protocol 8: e2894). For Nicotiana benthamiana, minimum 15 leaf disks per replicate were used for leaf disk tumor assay and minimum 5 leaf disks per replicate for transient assay (based on Anand et al. 2007, reference 22 in the manuscript).
Data exclusions	No data were excluded from this study.
Replication	All experiments were done in independent replicates as indicated in the respective figure legends.
Randomization	Samples were randomized in the growth chambers and greenhouses throughout the study
Blinding	Blinding was not used in these studies. Data reported are not subjective.

Reporting for specific materials, systems and methods

We require information from authors about some types of materials, experimental systems and methods used in many studies. Here, indicate whether each material, system or method listed is relevant to your study. If you are not sure if a list item applies to your research, read the appropriate section before selecting a response.

Materials & experimental systems

n/a	Involved in the study	
[ ]	[x]	Antibodies
[x]	[ ]	Eukaryotic cell lines
[x]	[ ]	Palaeontology and archaeology
[x]	[ ]	Animals and other organisms
[x]	[ ]	Human research participants
[x]	[ ]	Clinical data
[x]	[ ]	Dual use research of concern

Methods

n/a	Involved in the study	
[x]	[ ]	ChIP-seq
[x]	[ ]	Flow cytometry
[x]	[ ]	MRI-based neuroimaging

Antibodies

Antibodies used	PhiLOV antibody used in this study is a gift from John M. Christie of University of Glasgow.
Validation	PhiLOV antibody has been used previously (Gawthorne et al. 2016, Applied and Environmental Microbiology 82: 2700 - 2708; Buckley et al. 2016, Scientific Reports 6: 23463.).
